# Leptin-signaling inhibition results in efficient anti-tumor activity in estrogen receptor positive or negative breast cancer

**DOI:** 10.1186/bcr2321

**Published:** 2009-06-16

**Authors:** Ruben Rene Gonzalez, Amber Watters, Yanbo Xu, Udai P Singh, David R Mann, Bo R Rueda, Manuel L Penichet

**Affiliations:** 1 Department of Microbiology, Biochemistry and Immunology, Morehouse School of Medicine, 720 Westview Drive SW, Atlanta, GA 30310, USA; 2 Vincent Center for Reproductive Biology, Massachusetts General Hospital, 55 Fruit Street Their 901, Boston, MA 02114, USA; 3 Boston Biomedical Research Institute, 64 Grove Street, Watertown, MA 02472, USA; 4 Department of Pathology, Microbiology and Immunology, University of South Carolina, School of Medicine, 6311 Garners Ferry Road, Columbia, SC 29208, USA; 5 Department of Physiology, Morehouse School of Medicine, 720 Westview Drive SW, Atlanta, GA 30310, USA; 6 Department of Obstetrics, Gynecology and Reproductive Biology, Harvard Medical School, 55 Fruit Street, Boston, MA 02115, USA; 7 Department of Surgery, Division of Surgical Oncology; Microbiology, Immunology, and Molecular Genetics, Jonnson Comprehensive Cancer Center, David Geffen School of Medicine, University of California, 650 Charles Young Drive South, Los Angeles, CA 90095, USA

## Abstract

**Introduction:**

We have shown previously that treatment with pegylated leptin peptide receptor antagonist 2 (PEG-LPrA2) reduced the expression of vascular endothelial growth factor (VEGF), vascular endothelial growth factor receptor type 2 (VEGFR2) and growth of 4T1-breast cancer (BC) in syngeneic mice. In this investigation, PEG-LPrA2 was used to evaluate whether the inhibition of leptin signaling has differential impact on the expression of pro-angiogenic and pro-proliferative molecules and growth of human estrogen receptor-positive (ER^+^) and estrogen receptor-negative (ER^-^) BC xenografts hosted by immunodeficient mice.

**Methods:**

To test the contribution of leptin signaling to BC growth and expression of leptin-targeted molecules, PEG-LPrA2 treatment was applied to severe immunodeficient mice hosting established ER^+ ^(MCF-7 cells; ovariectomized/supplemented with estradiol) and ER^- ^(MDA-MB231 cells) BC xenografts. To further assess leptin and PEG-LPrA2 effects on ER^+ ^and ER^- ^BC, the expression of VEGF and VEGFR2 (protein and mRNA) was investigated in cell cultures.

**Results:**

PEG-LPrA2 more effectively reduced the growth of ER^+ ^(>40-fold) than ER^- ^BC (twofold) and expression of pro-angiogenic (VEGF/VEGFR2, leptin/leptin receptor OB-R, and IL-1 receptor type I) and pro-proliferative molecules (proliferating cell nuclear antigen and cyclin D_1_) in ER^+ ^than in ER^- ^BC. Mouse tumor stroma in ER^+ ^BC expressed high levels of VEGF and leptin that was induced by leptin signaling. Leptin upregulated the transcriptional expression of VEGF/VEGFR2 in MCF-7 and MDA-MB231 cells.

**Conclusions:**

These results suggest that leptin signaling plays an important role in the growth of both ER^+ ^and ER^- ^BC that is associated with the leptin regulation of pro-angiogenic and pro-proliferative molecules. These data provide support for the potential use of leptin-signaling inhibition as a novel treatment for ER^+ ^and ER^- ^BC.

## Introduction

Leptin is a small nonglycosylated protein (16 kDa) product of the *ob *gene. White adipose tissue is the primary source of leptin in benign tissue, but leptin is also expressed and secreted by cancer cells [1]. Leptin exclusively binds to its receptor, OB-R. Several isoforms of OB-R are found in diverse tissues and in cancer cells including the long isoform, OB-Rb [2,3]. Upon leptin activation, the OB-R isoforms can utilize a number of diverse signaling pathways relevant to cancer growth [4,5]. The well-documented biological actions of leptin at the hypothalamic level occur through OB-Rb signals that are linked to the control of appetite and energy balance [4].

Evidence is mounting to support the idea that leptin is the link between obesity and the higher incidence of a variety of cancers [6,7]. Several studies show that conditions characterized by high levels of leptin (female gender, obesity, menopause) are positively correlated with a higher incidence of breast cancer (BC) [8-10]. For BC patients, obesity can be an indicator of a poor prognosis even after the administration of adjuvant chemotherapy [6,7]. Nevertheless, there are contradicting reports showing no association between serum levels of leptin in premenopausal or postmenopausal women and the risk of BC [11,12]. Leptin and OB-R levels, however, are higher in BC cells than in normal mammary cells [13,14].

Almost all BC cells can develop metastases. This depends on the intricate relations of numerous tumor cell factors that include location and extension of cancer, the type and differentiation of the tumor cells, as well as other only incompletely understood factors. A role for the mammary fat pad in mammary gland development and enhancement of the growth and ability to metastasize BC cells has been described. Cytokines, the tumor microenvironment, adipose tissue, and the tissue microenvironment in remote organs could contribute to prime BC cells, promoting metastasis. Among these factors, TNFα and IL-1 are potent leptin inducers in adipose tissue [15]. The majority of BC cells express estrogen receptor (ER) and their growth is mainly driven by ER signaling [16] that could also be activated by leptin signaling [17-19]. A high level of OB-R in BC is a significant risk factor, independent of ER expression and other risk factors [13]. Moreover, there is significant correlation between the levels of leptin/OB-R in BC and a higher incidence of BC metastatic disease, poor prognosis, and lower survival rate of BC patients [13,14]. Leptin signaling could play an important role in the growth of highly invasive, metastatic, and more deadly estrogen receptor-negative (ER^-^) BC cells that do not respond to endocrine therapy and are mostly treated with chemotherapeutics that exhibit several detrimental side effects [20].

Leptin's pleiotropic effects are linked to diverse processes that if de-regulated could contribute to the growth of cancer; that is, proliferation, anti-apoptosis, angiogenesis, extracellular membrane component changes and metastasis [21-24]. Leptin enhances the expression of cell cycle regulators cdk-2 and cyclin D_1 _in human BC cells [22,23] and of pro-angiogenic factors in mouse mammary [24] and endometrial cancer cells [3]. Furthermore, leptin signaling is related with an increase cell survival since it can upregulate the expression of the anti-apoptotic protein, Bcl-2 [25-27]. Leptin's pleiotropic actions may therefore impact the growth of cancer through a variety of mechanisms. Hence, leptin may play an important role in controlling the proliferation, survival, and migration of cells involved in cancer growth. A recent published study by Perera and colleagues has reinforced this idea, showing data supporting leptin promotion of mammary tumor (MT) growth through multiple mechanisms, including regulating the cell cycle, apoptosis, and modulating the extracellular environment [28]. Little is known, however, about the exact mechanism(s) by which leptin contributes to tumor progression.

Data from animal studies reinforced the idea that leptin can contribute to BC growth. Obese rodents have a higher incidence of MTs than lean controls [29]. In contrast, obese mice with deficiency of leptin-signaling (*ob/ob *and *db/db*) have a significantly lower incidence of MT than their lean littermates [30,31]. Furthermore, nonobese mouse mammary tumor virus/human transforming growth factor-alpha mice have a high rate of MT development [29], which is offset in the offspring when they are crossed with leptin-signaling-deficient mice [30,31]. We previously reported that the blockade of leptin actions in mice hosting syngeneic MTs delayed tumor onset and reduced tumor growth [24]. Well known, however, is the fact that BC has diverse genetic and phenotypic heterogeneity. No single animal model can therefore fully represent all of the possible pathways by which human BC develops or progress.

The aim of the present study was to evaluate the impact of leptin-signaling inhibition on the growth of human BC xenografts and their expression of leptin-targeted molecules. Based on the reported leptin-mediated overexpression of aromatase [18] and transactivation of ER [19] in MCF-7 cells, we hypothesized that the effects of pegylated leptin peptide receptor antagonist 2 (PEG-LPrA2) on the growth of human BC xenografts will be more evident in MCF-7 ER^+ ^BC than in MDA-MB231 ER^- ^BC. This study shows that leptin signaling plays an important role in the growth of both ER^+ ^and ER^- ^human BC xenografts; ER^+ ^BC cells, however, were more responsive to PEG-LPrA2 treatment. Leptin signaling regulates the expression of angiogenic and pro-proliferative factors in BC and tumor stroma. The leptin tumor-promoting effects are probably direct (cell proliferation and survival) and indirect via the regulation of molecules involved in tumor growth. Collectively, these results strongly suggest that inhibition of leptin signaling may have potential novel therapeutic value for controlling ER^+ ^and ER^- ^BC growth.

## Materials and methods

### Antibodies and reagents

Antibodies for human vascular endothelial growth factor (VEGF) (A-20), for vascular endothelial growth factor receptor type 2 (VEGFR2) (Flk-1 or KDR; A-3), for cyclin D_1 _(HD11), for human IL-1 receptor type I (IL-1R tI) (N-20, proliferating cell nuclear antigen (PCNA); FL-261), for ERα (MC-20), for leptin (Y-20), for human OB-Rb (long isoform intracellular COOH end; C-20) and for cytokeratin 8/18 (0.N.352), blocking peptide antibody for competition studies, positive controls, protein G-agarose and the rabbit/goat ABC staining kit were obtained from Santa Cruz Biotechnology, Inc. (Santa Cruz, CA, USA). β-Actin antibody (ab8226) was purchased from Abcam Inc. (Cambridge, MA, USA). RPMI-1640 medium was obtained from American Type Culture Collection (Manassas, VA, USA). Fetal bovine serum was obtained from Gemini Bioproducts (Woodland, CA, USA), and antibiotic-antimycotic mixtures were purchased from Gibco BRL Products (Gaithersburg, MD, USA). Succinimidyl propionate polyethylene glycol (20 kDa) was obtained from Nektar Therapeutics (Huntsville, AL, USA). Other chemicals were obtained from Sigma Inc. (St Louis, MO, USA).

### Leptin peptide receptor antagonist

Leptin receptor antagonist 2 and a scrambled peptide for control were synthesized and purified as described elsewhere [32]. To increase their bioavailability, the peptides were covalently bound to succinimidyl propionate polyethylene glycol (20 kDa; half-life > 60 hours) at 5:1 molecular ratios (leptin receptor antagonist 2:polyethylene glycol) in PBS (pH 7.0), following the manufacturer's instructions (Nektar Therapeutics). In contrast to unconjugated leptin receptor antagonist peptides, their polyethylene glycol derivatives are water-soluble.

### Cell culture

The human BC adenocarcinoma cell lines MCF-7 (ER^+^) and MDA-MB231 (ER^-^) (American Type Culture Collection) were cultured (1.5 × 10^5 ^cells/well; duplicate wells; experiments repeated, n = 3) on uncoated flat-bottomed plastic 12-well plates with complete growth medium (American Type Culture Collection). Semi-confluent cells were cultured for 48 hours in basal medium (without fetal bovine serum) containing leptin (0, 0.6, 1.2 and 6.25 nM, equivalent to 0, 10, 20, and 100 ng/ml) and/or PEG-LPrA2 and inert control, pegylated scrambled peptide (Sc-PEG) (5 mM dissolved in water; final concentration in the medium, 3 μM). Conditioned media were harvested and cells were lysed as described elsewhere [24]. Protein concentrations were determined by the Bio-Rad kit (Bio-Rad Lab., Hercules, CA, USA).

### Real-time RT-PCR

Total RNA was extracted, purified from cells (RNeasy and RNase-Free DNase Set; Qiagen Inc., Valencia, CA, USA) and quantified (Quanti-iT RNA Assay Kit/Qubit fluorometer; Invitrogen, Carlsbad, CA, USA). cDNA was synthesized using the iScript cDNA kit (Bio-Rad) and a control without RT was used for each reaction to exclude chromosomal DNA contamination. cDNA samples were analyzed by real-time PCR using IQ SYBR Green Supermix (Bio-Rad). Relative expression values *R *were calculated using 18S rRNA as reference (n = 3):



The sequences of primers for human VEGF mRNA (180 bp DNA fragment), VEGFR2 (Flk1) mRNA (114 bp fragment) and 18S rRNA (317 bp DNA fragment) are available upon request. The PCR conditions were as follows: one cycle, 95°C for 3 minutes; and 45 cycles, 95°C for 30 seconds, 52°C for 30 seconds and 72°C for 30 seconds.

### Therapeutic treatment to mice hosting established breast cancer xenografts

All animal studies were approved by the Morehouse School of Medicine Institutional Review Board. Ovariectomized and nonovariectomized NOD.CB17-Prkdcscid/NCrCrl (SCID) mice, 6 weeks old, were obtained from Charles River Laboratories (Wilmington, MA, USA). Ovariectomized mice were subcutaneously implanted with an estradiol capsule (2 mg 17β-estradiol and 1.6 mg cholesterol) that was replaced every 21 days to sustain the growth of ER^+ ^BC cells. MCF-7 cells for ovariectomized mice or MDA-MB231 cells for nonovariectomized mice (2 × 10^6 ^cells/matrigel 4 mg/ml) were orthotopically inoculated into the mammary fat pads of mice (second row, right nipple). The tumor take rate was 100% in all experiments.

Before treatment, the ovariectomized mice hosting MCF-7 BC were slightly heavier than those nonovariectomized mice hosting MDA-MB231. Once tumors reached an approximate volume of 100 mm^3 ^(measured with a caliper; π/6 × width^2 ^× length), 10 mice/group hosting MCF-7 ER^+ ^BC and MDA-MB231 ER^- ^BC were randomly allocated to two groups per tumor type such that their tumor size and body weight were similar. The mice were then treated with PEG-LPrA2 for leptin-signaling inhibition or with inert Sc-PEG for controls, both 50 μl/0.5 mM in PBS every 48 hours, given by intravenous injection. Because the PEG-LPrA2 half-life is about 60 hours, this schedule will ensure a continuous plasma level of the antagonist. Treatments ended after 18 days. After 8 hours of fasting and before euthanasia, blood was drawn from the retro-orbital vein of mice (mild anesthesia; 400 μl avertin, 200 mg/kg body weight) for VEGF and leptin determinations [24].

### Mammary tumor growth

Changes in MT size were determined by caliper measurements on a weekly basis. Tumors were dissected and weighed after euthanasia. The impact of treatment on general health, body weight, and food intake was recorded weekly. Carcass weight was determined post mortem.

### Leptin targets in mammary tumors

#### Immunoprecipitation/western blot

The levels of VEGF, VEGFR2, OB-Rb, cyclin D_1_, and PCNA in tumor lysates were determined by immunoprecipitation/western blot. Briefly, protein concentrations in lysates from cell cultures and MTs were determined by the Bradford method (Bio-Rad). Thirty micrograms of protein were analyzed by western blot either directly or after immunoprecipitation with protein A or protein G-agarose beads following the manufacturer's instructions (Pierce, Rockford, IL, USA). Antibodies against β-actin were used as loading controls. Nonspecific mouse, rabbit, and goat IgGs were used as negative controls for western blot analysis. For quantitative evaluation of antigen expression, the blots were scanned and analyzed by the NIH Image program [24,33,34].

#### Immunohistochemistry

To assess the potential effects of blockade of OB-R function *in vivo *on the expression of various antigens (that is, VEGF, VEGFR2, OB-Rb, leptin, IL-1R tI, PCNA, and cyclin D_1_), immunohistochemistry in paraffin block sections (4 μm) was performed. Negative controls were also included. Briefly, unmasking of tissue antigens was performed by heat treatment in sodium citrate buffer (pH 6, 10 mM) at 95°C for 15 minutes and partial digestion at 37°C for 10 minutes with protease (Sigma Inc.). After quenching endogenous peroxidase activity with H_2_O_2 _(3% water solution) and blocking (2.5% horse or rabbit normal serum), tissue sections were incubated for 1 hour at room temperature with the following primary antibodies diluted in PBS- 0.1% BSA: anti-VEGF, anti-VEGFR2, anti-OB-Rb, anti-leptin, anti-IL-1R tI, anti-PCNA, and anti-cyclin D_1 _(all at 1 μg/ml). Monoclonal antibody against cytokeratin 8/18 (dilution 1:100) was routinely used to detect tumor epithelial components. Biotinylated secondary antibodies were used. The tissues were incubated with a streptavidin-biotin-peroxidase system according to the manufacturer's directions (Vectastain, ABC-AP kit; Vector, Burlingame, CA, USA), counterstained with hematoxylin (Dako Corp., Carpinteria, CA, USA), and mounted with VectaMount (Vector). Negative controls included tissue preparations in which the primary antibodies were substituted by irrelevant species-matched IgG. Negative controls for competitive studies with primary antibodies were generated by reincubation with their respective blocking peptides (20 μg/ml; Santa Cruz Biotechnology). All washing steps were performed by immersion of the preparations three times in PBS for 5 minutes at room temperature [24,34].

### Vascular endothelial growth factor and leptin concentrations

The levels of human and mouse VEGF and leptin in conditioned media from cell cultures, mouse sera, and MT lysates were determined by ELISA (R&D Systems Inc., Minneapolis, MN, USA).

### Statistical analysis

A one-way analysis of variance test with Dunnett error protection and confidence interval of 95% was used from the Analyse-it for Microsoft Excel (Leeds, UK) [35] analysis of *in vivo *and *in vitro *treatments. The experiments were repeated (n = 3) and all samples were analyzed in duplicate. The data were expressed as mean ± standard error. *P *< 0.05 was considered statistically significant. The model included the main effects of treatments and replicates.

## Results

### ER^+ ^and ER^- ^breast cancer xenograft growth

**Figure 1:**
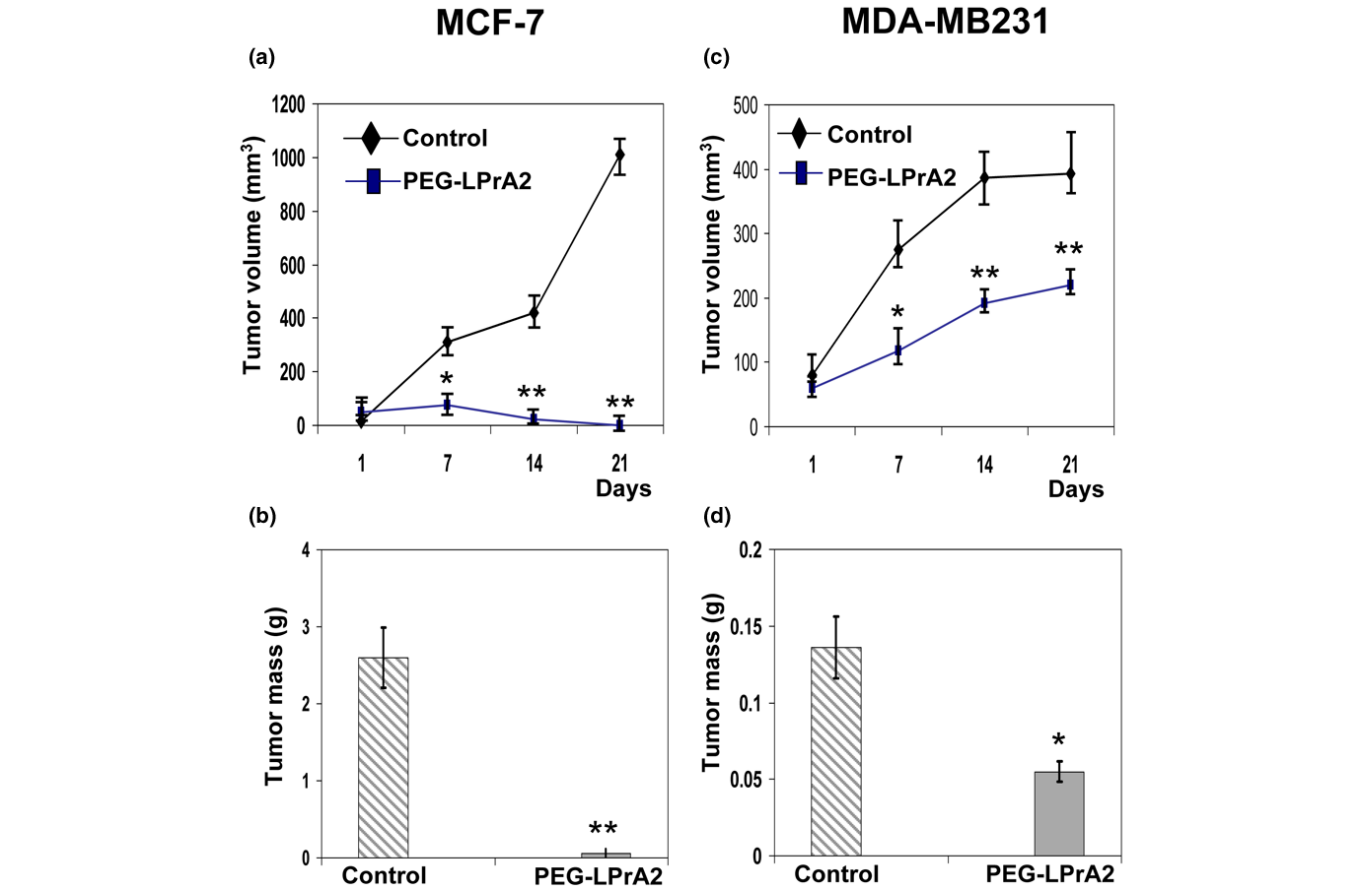
Impact of PEG-LPrA2 treatment on growth of estrogen receptor positive and negative breast cancer xenografts. **(a) **Growth of established MCF-7 breast cancer (BC) xenografts. **(b) **Tumor mass of established MCF-7 BC xenografts. **(c) **Growth of established MDA-MB231 BC xenografts. **(d) **Tumor mass of established MDA-MB231 BC xenografts. Female SCID mice were orthotopically inoculated into the mammary glands with human estrogen-receptor-positive MCF-7 (for ovariectomized mice) or estrogen-receptor-negative MDA-MB231 cells (2 × 10^6^). The mice were treated with pegylated leptin peptide receptor antagonist 2 (PEG-LPrA2) (n = 10/each cell type) or with pegylated scrambled peptide (control; n = 10/each cell type). Ovariectomized mice were supplemented with a subdermal estradiol capsule. Tumor growth was determined using a caliper (tumor volume = π/6 × width^2 ^× length). Data expressed as mean ± standard error. *P < 0.05 and **P < 0.01, significant differences with respect to control mice.

Injection of PEG-LPrA2 into mice with established MCF-7 BC xenografts resulted in a dramatic reduction (>40-fold) in the growth of tumor explants (Figure 1a). The reduction of MCF-7 tumors by PEG-LPrA2 was significant after 1 week of treatment. Moreover, at the end of treatment (18 days) the tumors were not palpable, which was confirmed after euthanasia and dissection of the tumor area. This was in contrast to those mice receiving control solution, Sc-PEG (Figure 1b). The treatment with PEG-LPrA2 was also effective in reducing MDA-MB231 BC xenograft growth. Mice hosting established MDA-MB231 BC had a significant reduction of tumor growth rate (Figure 1c). At week 3 of PEG-LPrA2-treatment BC, the volume of MDA-MB231 tumors was decreased approximately twofold when compared with the control mice (Figure 1c), which was assessed by a decrease in mass (Figure 1d). MCF-7 tumor masses from control mice were bigger than those from MDA-MB231 tumors. This could be due to increased vascularization in MCF-7 BC in control mice. The MCF-7 tumors were less differentiated (less prominent nucleoli, less intense chromatin change patterns, non-uniform large cells encountered together with small cells, and so forth) than those from MDA-MB231 BC.

### Leptin-targeted molecules in breast cancer xenografts

**Figure 2:**
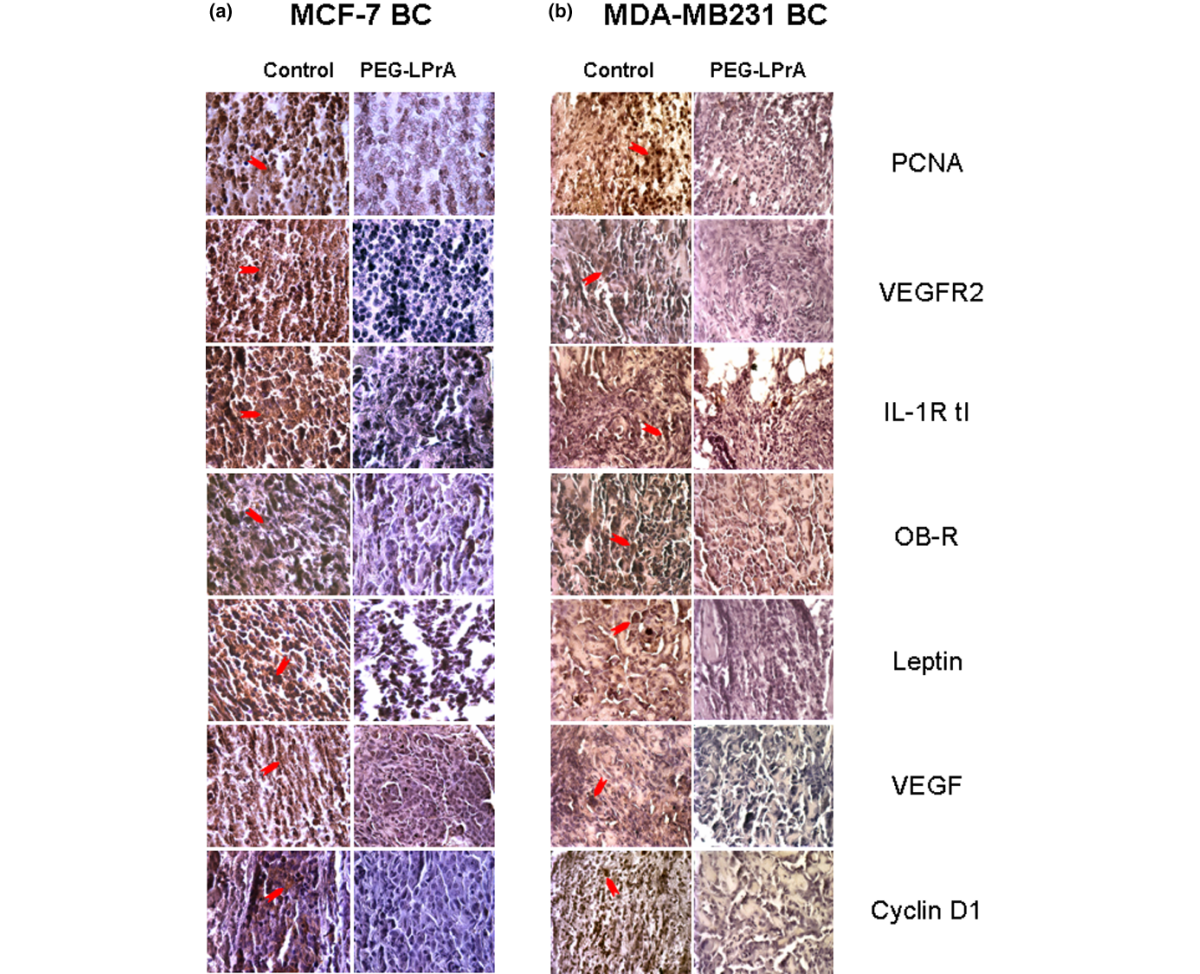
Proliferation, angiogenesis, and inflammation-related molecules in estrogen receptor positive and negative breast cancer xenografts. **(a) **Estrogen-receptor-positive MCF-7 breast cancer (BC) and **(b) **estrogen-receptor-negative MDA-MB231 BC from control (pegylated scrambled peptide) and pegylated leptin peptide receptor antagonist 2 (PEG-LPrA2)-treated SCID mice. Pictures show representative results from immunohistochemical analysis of pro-proliferative and pro-angiogenic molecules (n = 5; magnification x100). Arrows indicate stronger staining of the antigens in tumors from controls than tumors from PEG-LPrA2-treated mice. IL-1R tI, interleukin-1 receptor type I; OB-R, leptin receptor; PCNA, proliferating cell nuclear antigen; VEGF, vascular endothelial growth factor; VEGFR2, vascular endothelial growth factor receptor type 2.

Immunohistochemistry analysis revealed that the levels of leptin, OB-R, human VEGF, VEGFR2 or Flk-1, PCNA, human IL-1R tI and cyclin D_1 _were significantly lower in MCF-7 tumors from mice treated with PEG-LPrA2 than in tumors from Sc-PEG-treated controls (Figure 2a). Similar results were found in MDA-MB231-derived MTs from mice treated with PEG-LPrA2 (Figure 2b). Reduction in the expression of these antigens was more evident, however, in MCF-7 BC than in MDA-MB231 BC (Figure 2).

**Figure 3:**
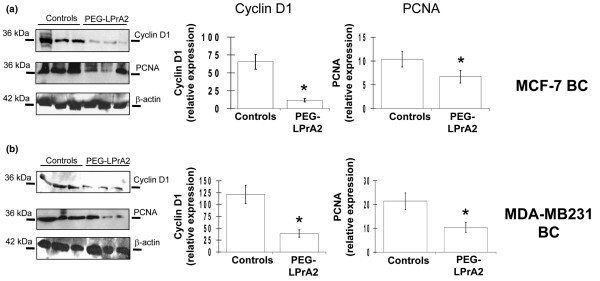
Effects of leptin inhibition on levels of proliferating cell nuclear antigen and cyclin D_1_. **(a) **MCF-7 breast cancer (BC) xenograft and **(b) **MDA-MB231 BC xenograft western blot results for proliferating cell nuclear antigen (PCNA) and cyclin D_1 _in tumor lysates from controls and SCID mice treated with leptin signaling antagonist (pegylated leptin peptide receptor antagonist 2 (PEG-LPrA2)). β-Actin was used as a loading control for western blot analysis. *P < 0.05, significant difference in mice treated with PEG-LPrA2 with respect to control mice receiving pegylated scrambled peptide. Data (mean ± standard error) show representative results (n = 10 tumors/treatment).

The impact of PEG-LPrA2 treatment on proliferation markers (PCNA and cyclin D_1_) was further investigated in tumor lysates by western blot analysis (Figure 3). A significant reduction of PCNA and cyclin D_1 _levels was found in MCF-7 BC (Figure 3a) and MDA-MB231 BC (Figure 3b) from mice treated with PEG-LPrA2.

### VEGF/VEGFR2 and leptin/OB-Rb in breast cancer xenografts

**Figure 4:**
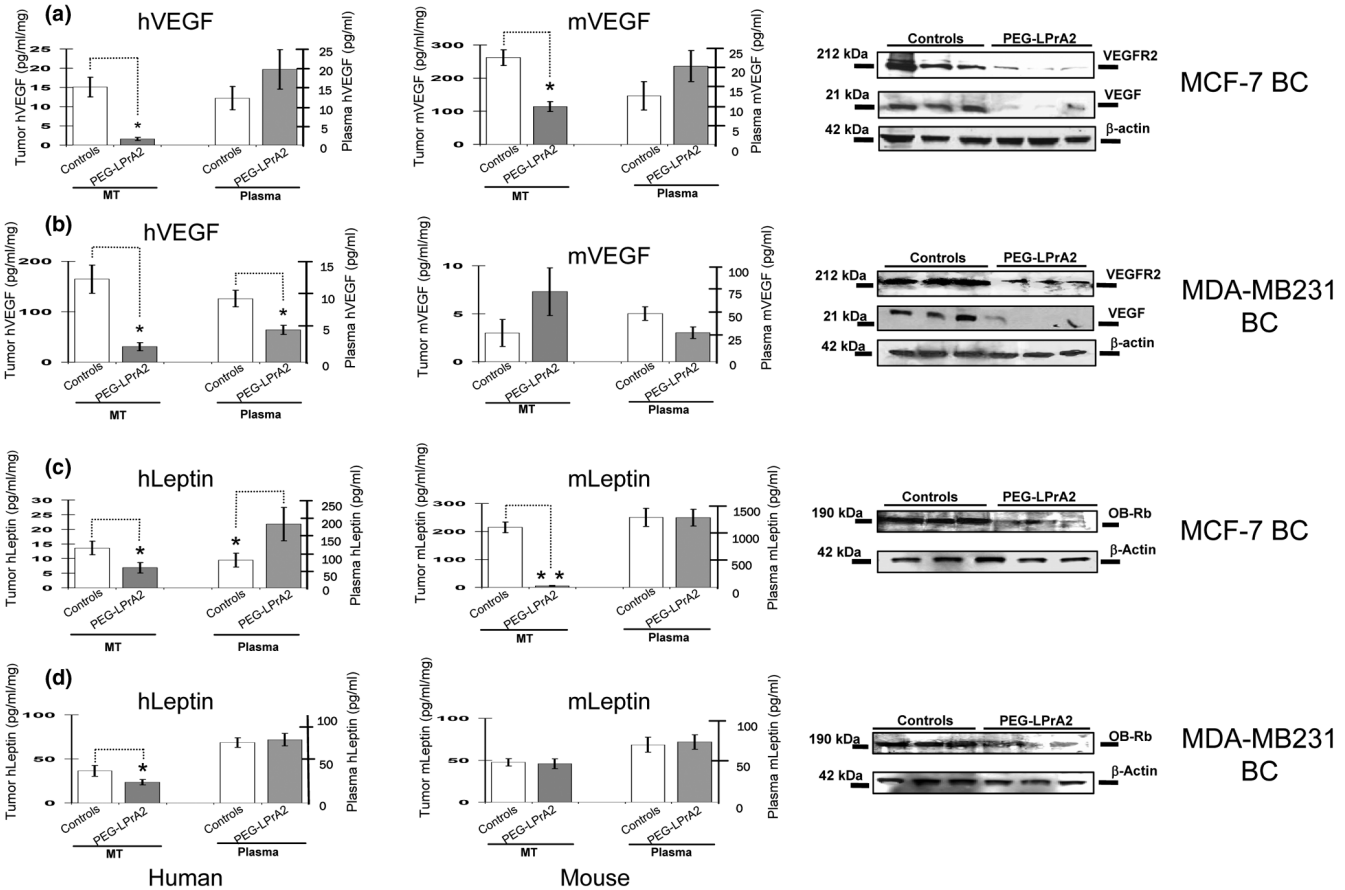
Leptin-inhibition effects on levels of human and mouse VEGF/VEGFR2 and leptin/OB-Rb in SCID mice. Human and mouse vascular endothelial growth factor (hVEGF and mVEGF) and human and mouse leptin (hLeptin and mLeptin) concentrations in tumor lysates and plasma, and western blot results for human VEGF and vascular endothelial growth factor receptor type 2 (VEGFR2) (mouse and human) and for human leptin receptor (OB-Rb) in MCF-7 breast cancer (BC) (**a,c**) and MDA-MB231 BC (**b,d**), respectively. β-Actin was used as a loading control for western blot analysis. *P < 0.05 and **P < 0.01, significant difference in mice treated with pegylated leptin peptide receptor antagonist 2 (PEG-LPrA2) with respect to control mice receiving pegylated scrambled peptide, respectively. Data (mean ± standard error) show representative results (n = 10 tumors/treatment). MT, mammary tumor.

PEG-LPrA2 treatment significantly decreased the levels of human VEGF (~15-fold) in MCF-7 BC lysates (Figure 4a). Plasma levels of human VEGF were low and no significant differences were detected between PEG-LPrA2-treated mice hosting MCF-7 BC when compared with controls (Figure 4a). Levels of mouse VEGF in MCF-7 BC lysates from control mice were significantly higher than those for human VEGF. In contrast, levels of mouse VEGF in plasma from mice hosting MCF-7 BC and treated with PEG-LPrA2 were no different from those from control mice (Figure 4a). Immunoprecipitation/western blot analysis showed that VEGF and VEGFR2 levels were significantly reduced by PEG-LPrA2 in MCF-7 BC when compared with controls (Figure 4a).

Reduced levels of human VEGF were found in the MDA-MB231 BC from mice treated with PEG-LPrA2 (Figure 4b). Immunoprecipitation/western blot analysis showed that PEG-LPrA2 treatment reduced the levels of VEGFR2 in MDA-MB231 BC (approximately threefold; Figure 4b). In comparison, human VEGF levels in tumor lysates from control mice hosting MDA-MB231 BC xenografts were significantly higher (Figure 4b) than in control mice hosting MCF-7 BC xenografts (Figure 4a). In contrast, stroma in MDA-MB2231 BC from control mice had lower levels of mouse VEGF (Figure 4b) compared with stroma from MCF-7 BC xenografts hosted by control mice (Figure 4a).

PEG-LPrA2 treatment reduced the levels of human leptin in tumors from mice hosting MCF-7 BC xenografts (Figure 4c). Similarly, the levels of mouse leptin from mice treated with PEG-LPrA2 were lower than those from control but no differences were detected in plasma (Figure 4c). In a similar way, PEG-LPrA2 treatment reduced the levels of human leptin within MDA-MB231 BC xenografts but tumor levels of mouse leptin were similar between treated and controls (Figure 4d). In comparison, MDA-MB231 BC lysates from control mice exhibited higher levels of human leptin but lower levels of mouse leptin (Figure 4d) than those from control mice hosting MCF-7 BC xenografts (Figure 4c). Immunoprecipitation/western blot analysis revealed a notable reduction of human OB-Rb expression in MCF-7 and MDA-MB231 BC xenografts in mice treated with PEG-LPrA2 compared with controls (Figure 4c,d).

### General effects of PEG-LPrA2

Overall, PEG-LPrA2 was not toxic and did not affect the energy balance (body weight, carcass weight or food intake) of treated mice when compared with control mice hosting MCF-7 or MDA-MB231 BC xenografts. Significant differences were found, however, between carcass weights from mice hosting MCF-7 BC and MDA-MB231 BC (Table 1). An increased amount of abdominal fat was found in ovariectomized mice hosting MCF-7 BC xenografts compared with those hosting MDA-MB231 BC xenografts. These differences were found not related to PEG-LPrA2 or control treatments. PEG-LPrA2 delayed tumor onset and growth, and negatively impacted on the levels of pro-angiogenic, pro-inflammatory and pro-proliferative molecules in both types of BC. There were more notable effects of PEG-LPrA2, however, in mice hosting MCF-7-derived BC xenografts.

**Table 1:** Treatment effects on mouse energy balance and growth of MCF-7 and MDA-MB231 breast cancer xenografts

	MCF-7 (estrogen-receptor-positive) xenografts		MDA-MB231 (estrogen-receptor-negative) xenografts	
	
	PEG-LPrA2	Sc-PEG	PEG-LPrA2	Sc-PEG
Initial food intake (g/day)	2.2 ± 0.7	2.2 ± 0.4	2.6 ± 0.6	2.4 ± 0.2
Final food intake (g/day)	2.9 ± 0.5	2.8 ± 0.4	2.9 ± 0.6	2.9 ± 0.4
Food intake change (g/day)	+0.7	+0.6	+0.3	+0.5
Initial body weight (g)	19.7 ± 2.0	19.6 ± 0.7	18.8 ± 0.7	19.2 ± 0.3
Final body weight (g)	23.9 ± 0.7	24.9 ± 1.3	19.3 ± 1.3	20.0 ± 1.4
Body weight change (g)	+4.2	+5.3	+0.5	+0.8
Carcass weight (g)	17.1 ± 1.0	17.9 ± 0.7	14.2 ± 1.2*	14.6 ± 0.2*
Final tumor volume (mm^3^)	20.5 ± 5.0	>1,100 ± 180	210 ± 29	430 ± 45
*n*	10	10	10	10

Data expressed as mean ± standard deviation (n = 10 tumors/breast cancer type/treatment). *P < 0.05, significant difference in carcass weight of mice hosting MDA-MB-231 breast cancer xenografts treated with pegylated leptin peptide receptor antagonist 2 (PEG-LPrA2) or pegylated scrambled peptide (Sc-PEG) with respect to mice hosting MCF-7 breast cancer xenografts (n = 10 tumors/treatment).

### Leptin receptor and estrogen receptor in cancer cell cultures

MCF-7 and MDA-MB231 cells expressed OB-R, but only MCF-7 cells expressed ERα (immunocytochemistry and western blot; data not shown).

### Leptin in cancer cell cultures

MDA-MB231 cells (9.6 pg/ml/mg, equivalent to 0.41 pM) under basal conditions secreted more leptin (approximately fourfold) than MCF-7 cells (2.6 pg/ml/mg, equivalent to 0.15 pM).

### VEGF and VEGFR2 in cancer cell cultures

**Figure 5:**
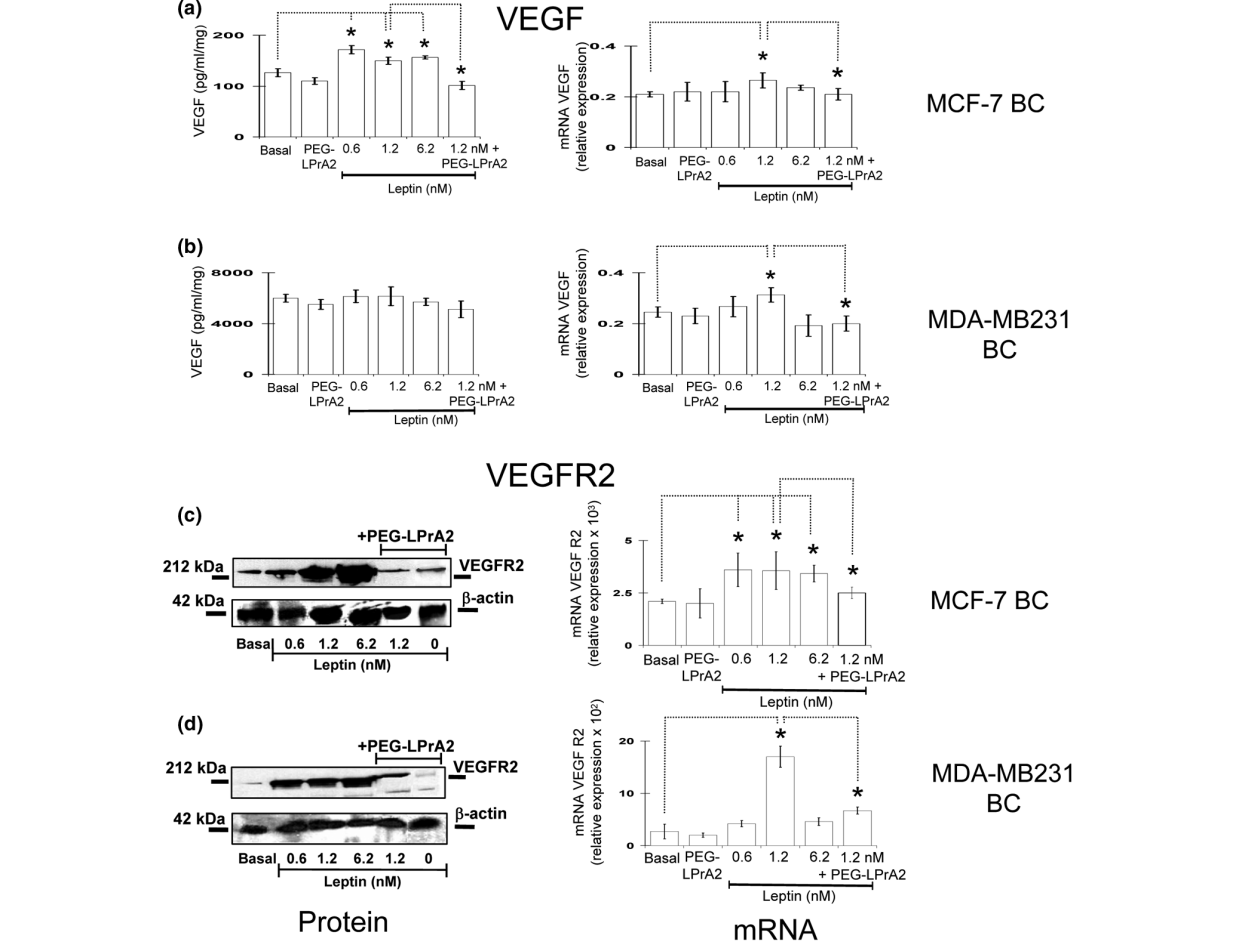
Leptin effects on VEGF and VEGFR2 protein and mRNA in MCF-7 and MDA-MB231 cultures. Vascular endothelial growth factor (VEGF) protein and mRNA and vascular endothelial growth factor receptor type 2 (VEGFR2) protein and mRNA levels in MCF-7 breast cancer (BC) (**a,c**) and MDA-MB231 BC cells (**b,d**), respectively. The cells were cultured for 48 hours in medium containing leptin (0 to 6.25 nM, equivalent to 10 to 100 ng/ml) and/or pegylated leptin peptide receptor antagonist 2 (PEG-LPrA2) (3 μM). Levels of VEGF protein in the supernatants determined by ELISA. Levels of VEGFR2 protein in cell lysates determined by immunoprecipitation/western blot. β-Actin used as loading control for western blot determinations. VEGF and VEGFR2 mRNA determined by real-time RT-PCR and expressed as relative values to basal conditions. All data were derived from a minimum of three independent experiments using different cell preparations. *P < 0.05, comparing levels of basal cells with treated cells.

Basal secretion of VEGF was much higher from MDA-MB231 (~46-fold; Figure 5b) than from MCF-7 (Figure 5a) cell cultures. Leptin significantly increased the levels of VEGF in medium of MCF-7 cell cultures at all the leptin doses assayed (Figure 5a) but had no effects on VEGF levels in MDA-MB231 cell cultures (Figure 5b). Importantly, the co-incubation of MCF-7 cells with leptin and PEG-LPrA2 completely abrogated the leptin-mediated increase in VEGF in the conditioned medium (Figure 5a). Real-time RT-PCR analysis indicated that leptin upregulates the transcriptional expression of VEGF in MCF-7 cells (Figure 5a) and in MDA-MB231 cells (Figure 5b). The co-incubation of cells with leptin and PEG-LPrA2 inhibited the leptin effects on VEGF mRNA levels. In both types of cells, leptin in a dose-response manner significantly increased the levels of VEGFR2, and PEG-LPrA2 abrogated these effects (Figure 5c,d). Real-time RT-PCR showed that leptin also increased the expression of VEGFR2 mRNA in both cell types (Figure 5c,d) that were inhibited by PEG-LPrA2.

## Discussion

The present investigation outlines for the first time the contributions of leptin to the growth of human ER^+ ^BC xenografts and the very aggressive ER^- ^BC hosted by SCID mice. A potent antagonist for the leptin receptor, PEG-LPrA2 (with an extended half-life of 60 hours), was used to test the impact of leptin signaling inhibition on the growth of BC and the expression of leptin-targeted molecules important for BC angiogenesis and proliferation. It is well known that while there are significant strengths in the use of this mouse model that does not reject human BC cells, there also are limitations – including the lack of the physiological immune reaction to the disturbance of leptin's inflammatory functions that may influence the prediction of a BC patient's response to this therapy. To have an idea of how human BC could respond to PEG-LPrA2, however, it was necessary to use human BC growing in SCID mice. The significance of leptin-induced signaling in the regulation of VEGF and VEGFR2 expression (at protein and mRNA levels) was also investigated in cultures of MCF-7 (ER^+^) and MDA-MB231 (ER^-^) BC cells.

The data presented support the potential translational use of PEG-LPrA2 for prevention and/or treatment of BC. Differential effects for leptin signaling on the growth of ER^+ ^and ER^- ^BC cells *in vitro *have been reported [36,37]. These effects could be related to leptin-induced levels of aromatase [17,18] and to ER transactivation [19]. Leptin was therefore expected to have stronger effects on the growth of ER^+ ^BC. To investigate this hypothesis, SCID mice hosting ER^+ ^MCF-7 and ER^- ^MDA-MB231 BC xenografts were treated with a leptin-signaling antagonist, PEG-LPrA2.

An impressive reduction of growth of ER^+ ^and ER^- ^BC xenografts was found after PEG-LPrA2 treatment. Moreover, both types of human BC xenografts responded to PEG-LPrA2 treatment by reducing the expression of several leptin-targeted molecules. Different growth rates for MCF-7 BC and MDA-MB231 BC xenografts in control mice (receiving Sc-PEG) were detected after 14 days of treatment. Although the exact reasons for this finding are unknown, it could be related to the boost of estradiol after re-emplacing the estradiol capsules in ovariectomized mice hosting MCF-7 BC.

The present data further support the idea that leptin signaling plays an important role in BC development and/or progression that may be mechanistically linked to leptin-mediated upregulation of pro-angiogenic and pro-proliferative factors. Inhibition of leptin signaling, however, more markedly reduced the growth and expression of leptin-related molecules in MCF-7 BC in comparison with MDA-MB231 BC xenografts. Specifically, leptin signaling inhibition decreased the levels of VEGF and leptin and their respective receptors within both BC xenograft types. MDA-MB231 BC xenografts had higher levels of VEGF and leptin than MCF-7 BC xenografts. The inhibition of leptin signaling therefore almost completely blocked VEGF expression and reduced leptin levels within MCF-7 BC. Reasons for the relative high levels of human leptin found in plasma of treated mice hosting MCF-7 are unknown but could be related to cross-reactivity of ELISA antibodies with leptin peptide receptor antagonist 2 (composed by a stretch the human leptin molecule). The ovariectomized mice hosting MCF-7 showed higher accumulation of abdominal fat than those nonovariectomized hosting MDA-MB231 BC cells. PEG-LPrA2 has an enhanced half-life (60 hours) compared with leptin (approximately 1 hour). Preliminary pharmacokinetics studies showed that PEG-LPrA2 was found at a higher concentration in mouse adipose tissue (RR Gonzalez, unpublished data).

Leptin is an upstream regulator of angiogenic molecules [21,24,32,33,38-40]. Leptin peptide receptor antagonist 2 treatment decreased the levels of leptin-induced angiogenic molecules in mouse tumors and human endometrial cancer cells [3,24]. These data suggest that the inhibition of leptin signaling negatively impacted tumor growth by decreasing leptin-induced expression of several factors implicated in epithelial cell proliferation, adhesion, inflammation and angiogenesis; for example, β_3_-integrin [38,41,42], metalloproteinases [43], leukemia inhibitory factor, leukemia inhibitory factor receptor, IL-1, IL-1 receptor (IL-1R tI), and IL-1 receptor antagonist [32,34,38-40]. Many genes regulated by leptin in MCF-7 cells are related to growth factors, cell cycle regulators, extracellular matrix, metastasis (that is, cyclin D, cyclin G, cyclin-dependent kinase 2, p21, p27, p16, connective tissue growth factor, villin 2, and basigin), and anti-apoptosis (BCL2 and surviving) [28]. In line with this notion, the mice treated with PEG-LPrA2 had diminished expression of VEGF/VEGFR2, OB-R, leptin, IL-1R tI, PCNA and cyclin D_1_.

Even though normal mammary cells express leptin and OB-R, abnormal high levels of leptin/OB-R expression are found in BC that is related to both the metastasis and lower survival rates [1,11,13]. The reduction of leptin and OB-R levels in ER^- ^BC and ER^+ ^BC after PEG-LPrA2 treatment could therefore negatively impact on leptin's actions within BC and may have a potential value as subrogate markers to assess the efficacy of leptin-signaling inhibition as a novel anticancer therapy.

It is known that tumor stroma (noncancerous cells; that is, fibroblasts, immune and endothelial cells) could play a role in promoting tumor growth [44]. To gain further insight into mechanisms of leptin-induced tumor growth and angiogenesis in BC, the levels of mouse VEGF and leptin in tumor stroma were investigated. Interestingly, stroma from MCF-7 BC xenografts had higher levels of mouse VEGF and leptin than those found in stroma from MDA-MB231 BC xenografts. These data were in contrast to the higher levels of human VEGF secreted by MDA-MB231 BC in comparison with VEGF levels secreted by MCF-7 BC xenografts. These data suggest that MCF-7 ER^+ ^BC differentially secrete factors inducing the expression of VEGF and leptin by tumor stroma compared with MDA-MB231 BC. Factors secreted by MCF-7 BC could further promote via tumor-stroma angiogenesis and, consequently, tumor growth [44]. Remarkably, PEG-LPrA2 treatment decreased levels of human and mouse VEGF and leptin in MCF-7 ER^+ ^BC. This could explain in part the higher effectiveness of PEG-LPrA2 treatment in reducing tumor growth in MCF-7 ER^+^. Importantly, PEG-LPrA2 treatment did not affect mouse leptin levels in plasma, suggesting that this compound did not interfere with the systemic leptin metabolism. Indeed, no significant effects on body weight or carcass weight were found between mice hosting the same type of BC xenografts and treated with PEG-LPrA2 or Sc-PEG control.

The role of leptin signaling in the regulation of VEGF and VEGFR2 by MCF-7 and MDA-MB231 cancer cells was further studied *in vitro*. Leptin and VEGF basal levels were higher in MDA-MB231 compared with those from MCF-7 cells, but leptin increased the levels of secreted protein VEGF only in MCF-7 cells. In contrast, VEGF mRNA expression was upregulated by leptin in both cell types. This is probably due to the constitutive expression of VEGF and/or the autocrine and paracrine actions of leptin in MDA-MB231 cells. Remarkably, leptin-induced effects on VEGF and VEGFR2 levels were abrogated by co-incubation of cells with PEG-LPrA2. Data from *in vitro *studies correlate with findings from BC xenografts. These data further suggest that leptin could induce VEGF/VEGFR2 expression in BC.

Taken together, the present data support a role for leptin as a tumor growth factor. Moreover, the leptin effect is probably mediated via several angiogenic and pro-proliferative molecules in BC. The increased susceptibility of ER^+ ^BC compared with ER^- ^BC to the negative impact of PEG-LPrA2 leptin-signaling inhibition could partially be related to differential mechanisms for leptin regulation of VEGF/VEGFR2 in the BC cells used. In addition, it cannot be ruled out that leptin-signaling crosstalk could activate other signaling mechanisms related to essential factors for BC growth; that is, insulin-like growth factor [45] and human epidermal growth factor receptor-2 (HER2/*neu*; erbB2) [46]. HER2/*neu *increases the levels of the anti-apoptotic protein Bcl-2 [47] and pro-proliferative molecules, the cyclin-dependent kinase inhibitor p27Kip1 and the cell cycle regulatory protein cyclin D_1 _[48]. HER2/neu shows moderate to low expression by MCF-7 and MDA-MB231 [46], but the HER2/neu-leptin signaling link can further increase the levels of pro-survival factors [26,27] and pro-proliferative factors [24]. Off-target effects of PEG-LPrA2 treatment therefore cannot be excluded from the interpretation of data. Nevertheless, these data suggest that targeting leptin/OB-R functions may negatively impact tumor growth, angiogenesis, apoptosis, and expression of inflammation-related factors and could impair leptin-growth promoter-factor crosstalk in human BC.

Our present findings may have particular importance for designing new therapies for BC and other cancer types (that is, endometrial cancer, colon cancer, prostate cancer, and so forth) where obesity and leptin signaling have also been related to their incidence and growth [8,23]. Inhibition of leptin signaling could be especially useful for the treatment of more aggressive and invasive ER^- ^BC that is currently treated with a variety of chemotherapeutics with many debilitating side effects [20]. In contrast, preliminary pharmacokinetic and toxicological studies suggest that the high-molecular-weight PEG-LPrA2 derivative does not travel through the blood-brain barrier and therefore it is not bound to hypothalamic OB-R or accumulated in the central nervous system of mice. This suggests PEG-LPrA2 may not interfere with leptin biological actions at the hypothalamic level. PEG-LPrA2 shows no detrimental effects on the general health status of mice. Indeed, mice treated for more than 2 months with PEG-LPrA2 showed no evident toxicity or change in appetite/energy balance, insulin/glucose levels, or general health status (RR Gonzalez, unpublished data).

## Conclusions

Results from the present investigation strongly support the idea that leptin signaling plays an important role in the establishment and growth of both human ER^+ ^MCF-7 and ER^- ^MDA-MB231 BC xenografts. Leptin actions in these tumors are probably related to leptin-mediated increase in levels of VEGF/VEGFR2 and OB-R and other leptin-targeted molecules essential to BC growth. ER^+ ^BC was more responsive, however, to PEG-LPrA2 mediated inhibition of leptin signaling than ER^- ^BC. Overall, our data open the possibility that inhibition of leptin signaling may serve as a novel adjuvant for prevention and treatment of BC, particularly in populations under higher risk and exhibiting higher levels of leptin: such as obese and postmenopausal women. The alarming increase of incidence of obesity in the western countries emphasizes the importance of our findings on leptin-signaling inhibition for reduction of ER^+ ^BC and ER^- ^BC growth.

## Abbreviations

BC: breast cancer; bp: base pairs; BSA: bovine serum albumin; ELISA: enzyme-linked immunosorbent assay; ER: estrogen receptor; IL: interleukin-1; IL-1R tI: interleukin-1 receptor type I; MT: mammary tumor; OB-R: leptin receptor; PBS: phosphate-buffered saline; PCNA: proliferating cell nuclear antigen; PEG-LPrA2: pegylated leptin peptide receptor antagonist 2; SCID: severely compromised immunodeficient; Sc-PEG: pegylated scrambled peptide; TNF: tumor necrosis factor; VEGF: vascular endothelial growth factor; VEGFR2: vascular endothelial growth factor receptor type 2 (Flk-1).

## Competing interests

The authors declare that they have no competing interests. RRG is an inventor of the Boston Biomedical Research Institute's patent Leptin Peptide Antagonists (US Patent 7407929, Application No. 10/841,218; International Application No. PCT/US 05/15198). No financial benefits have been derived from this patent.

## Authors' contributions

RRG conceived of the study, participated in its design and coordination, and drafted the manuscript. AW participated in the animal trials, and helped carry out the immunohistochemical studies and western blot and ELISA determinations of relevant molecules. YX carried out the molecular genetic studies. UPS participated in the immunohistochemical studies. DRM participated in drafting the manuscript. BRR participated in the design of the study and drafted the manuscript. MLP participated in the design of the study and drafted the manuscript. All authors read and approved the final manuscript.
